# ISEV RNA Workshop—New York City, October 1–2, 2012

**DOI:** 10.3402/jev.v1i0.19857

**Published:** 2012-10-19

**Authors:** Clotilde Théry

**Affiliations:** INSERM U932, Institut Curie, Paris, France

The workshop focused on extracellular vesicle RNA (evRNA). It was organized by the International Society for Extracellular Vesicles (ISEV: www.isev.org) in New York on 1–2 October 2012 and was a resounding success. Jan Lötvall (President of ISEV, Gothenburg University, Sweden) and Marca Wauben (ISEV Board Member, Utrecht University, The Netherlands) organized it in a very friendly way in a beautiful venue, all of which was ideal in promoting open and successful scientific exchanges. In total, 50 participants were selected from over 100 applications, based on their expertise in the field of extracellular RNA and/or vesicles (microvesicles, exosomes, ectosomes and others) over several years. Ten percent of the participants came from Australia/Asia, with the remainder equally represented from Europe and the Americas. In addition, a WebEx access to the PowerPoint presentations was set up. This allowed for approximately 50 additional ISEV members to participate remotely in the seminar.

All 33 of the submitted brief abstracts were presented as short 15 minutes presentations including discussion during six plenary sessions. Participants were then separated into three parallel round-table discussions each day, aimed at discussing the technical and theoretical issues raised in the field, specifically on the following questions: (1) extracellular vesicle (EV) isolation; (2) evRNA isolation; (3) evRNA analysis by hybridization arrays, qPCR; (4) evRNA analysis by deep sequencing and bioinformatics; (5) artificial RNA loading/engineered vesicles; and (6) analysis of functional transfer of evRNA. The conclusions and/or the major issues raised during each round-table group were discussed in plenary sessions at the end of each day.

Most discussed issues included (1) level of purification of EV preparations and the need to improve and share purification and separation protocols; (2) presence of RNA inside versus outside EVs and relevance of RNase treatment to address this issue; (3) absolute and relative amounts of mRNA, miRNA, and other non-coding RNA, including ribosomal RNA in EVs; (4) definite demonstration of a functional mRNA and/or miRNA transfer between cells of the same organisms via EVs (participants agreed that a transfer of virus-derived miRNA via infected cell-derived vesicles had been convincingly demonstrated).

From this, the participants will go on to coordinate their efforts to prepare several ISEV Position Papers, to be published in the *Journal of Extracellular Vesicles* (JEV), in particular for phrasing recommendations for purification of EVs and analysis of their RNA cargo.

As Secretary General of ISEV, I want to deeply thank Jan and Marca for their very efficient efforts to organize such an interactive and useful workshop. I also want to thank the other ISEV board members who attended this meeting and who participated in its smooth organization (Chris Gardiner, Yong Song Gho, Andrew Hill, Melissa Piper, Peter Quesenberry, Hidetoshi Tahara), Xandra Breakefield and Fred Hochberg who efficiently chaired two round-tables, and all the attendees for their eager and constructive participation.

This event will definitely help to improve the field, by guiding and enhancing investigators to achieve the best experimental quality in their research on EVs and evRNA.

Clotilde Théry
INSERM U932, Institut Curie, Paris, France
Email: clotilde.thery@urie.fr

